# Selenium, Iodine and Iron–Essential Trace Elements for Thyroid Hormone Synthesis and Metabolism

**DOI:** 10.3390/ijms24043393

**Published:** 2023-02-08

**Authors:** Josef Köhrle

**Affiliations:** Charité–Universitätsmedizin Berlin, Corporate Member of Freie Universität Berlin, Humboldt-Universität zu Berlin, Berlin Institute of Health, Institut für Experimentelle Endokrinologie, Max Rubner Center (MRC) for Cardiovascular Metabolic Renal Research, D-10115 Berlin, Germany; josef.koehrle@charite.de

**Keywords:** essential trace elements, selenium, iodine, iron, thyroid hormone system, deiodinases, thyroperoxidase, hemoprotein, glutathione peroxidase, thioredoxin reductase, SELENOP (selenoprotein P), gene variants, genetic predisposition, biomarker

## Abstract

The adequate availability and metabolism of three essential trace elements, iodine, selenium and iron, provide the basic requirements for the function and action of the thyroid hormone system in humans, vertebrate animals and their evolutionary precursors. Selenocysteine-containing proteins convey both cellular protection along with H_2_O_2_-dependent biosynthesis and the deiodinase-mediated (in-)activation of thyroid hormones, which is critical for their receptor-mediated mechanism of cellular action. Disbalances between the thyroidal content of these elements challenge the negative feedback regulation of the hypothalamus–pituitary–thyroid periphery axis, causing or facilitating common diseases related to disturbed thyroid hormone status such as autoimmune thyroid disease and metabolic disorders. Iodide is accumulated by the sodium-iodide-symporter NIS, and oxidized and incorporated into thyroglobulin by the hemoprotein thyroperoxidase, which requires local H_2_O_2_ as cofactor. The latter is generated by the dual oxidase system organized as ‘thyroxisome’ at the surface of the apical membrane facing the colloidal lumen of the thyroid follicles. Various selenoproteins expressed in thyrocytes defend the follicular structure and function against life-long exposure to H_2_O_2_ and reactive oxygen species derived therefrom. The pituitary hormone thyrotropin (TSH) stimulates all processes required for thyroid hormone synthesis and secretion and regulates thyrocyte growth, differentiation and function. Worldwide deficiencies of nutritional iodine, selenium and iron supply and the resulting endemic diseases are preventable with educational, societal and political measures.

## 1. Introduction

### 1.1. Thyroid Hormones and Trace Elements

TH-related non-contagious disorders, benign and malignant, cover a broad spectrum from common (autoimmunity-related hypothyroidism) to rare diseases (Graves’ disease and thyroid cancer) challenging the global health system and economy due to the necessary diagnostic, therapeutic and monitoring measures. L-Thyroxine (T_4_), the prohormone exclusively secreted by the thyroid gland, ranks among the top five drugs prescribed by physicians [1] (https://clincalc.com/DrugStats/Drugs/Levothyroxine, accessed on 6 February 2023). Various forms of thyroid cancer are the most frequent malignancies of the endocrine system [2]. While nutritional iodine (I) supply markedly improved during the last few decades since the first introduction of table salt iodination programs in Switzerland and USA a century ago, various regions and countries on our globe still have not yet installed such preventive programs, or those measures taken by government and health organizations are insufficient, not well implemented and monitored, or only rely on voluntary behavior (Germany) [3]. There has also been a decreasing production, distribution and consumption of iodinated table salt, especially in economically wealthy societies such as the European Union [4]. 

**Figure 1:**
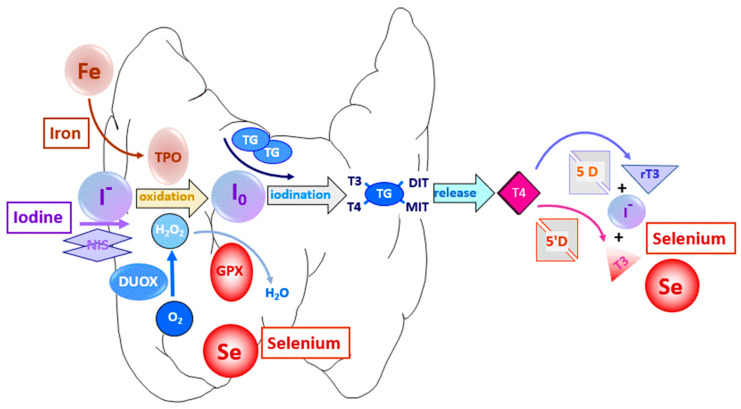
Essential role of the three trace elements iodine, selenium and iron in thyroid hormone biosynthesis and activation. Abbreviations: NIS: Na^+^-Iodide-Symporter (iodide uptake); DUOX: dual oxidase (H_2_O_2_ generation); TPO: thyroperoxidase (hemoprotein); TG: thyroglobulin (synthesis and storage protein); GPX: glutathione peroxidase (antioxidant defense); DIO: deiodinase (TH in-/activation).

While iodine’s status already receives international attention and awareness, public information of and preventive actions for adequate selenium (Se) supply are still in their infancy. Only a few years ago the human selenoproteome, encoded by 25 genes, was characterized (for review, see [5]), and not all functions of these proteins have been identified yet. However, the healthy human thyroid gland ranks very high in its relative and absolute selenium content and expresses most selenoproteins [6] (Figure 1). The three deiodinase (DIO; EC:1.21.99.4, EC:1.21.99.4) enzymes, key to the local cellular activation and the inactivation of TH T_4_ and T_3_ in the thyroid gland and target tissues of TH action, were identified 30 years ago as the second selenoprotein family after glutathione peroxidases [7]. Glutathione peroxidases (GPx) defend the thyroid against continuous H_2_O_2_ production [8], together with the thioredoxin reductase (TXNRD) families [9], as well as the various selenoproteins involved in cellular redox controls and the quality control of the biosynthesis of thyroglobulin (TG), the major product of thyroid follicles, an essential protein for TH biosynthesis, storage and secretion [10]. Se deficiency impairs thyroid function and TH production and secretion and various studies test whether and which thyroid-hormone-related disorders might be prevented or treated by (adjuvant) nutritional Se supply or Se-containing drugs [11]. 

More solidly established is the critical role of an adequate iron (Fe) status for TH biosynthesis and thyroid function [12] (Figure 1). An inadequate Fe supply, widely prevalent in less affluent regions and societies of the Global South, impairs the efficiency of TH biosynthesis even with an adequate iodine supply. Females in their reproductive age are especially vulnerable to this constellation considering their monthly iron losses during menstruation. Whether and to what extent this physiological factor contributes to the higher prevalence of benign and malignant thyroid-hormone-related diseases in females is the subject of ongoing research [13]. Apart from the globally diverse picture of nutritional deficiencies for thyroid trace element supply, accumulating information on the role of a genetic predisposition and polymorphic variations in the individual genetic makeup of the components of the thyroid hormone system (THS) shows the need for taking a new path towards a more personalized approach in preventive and therapeutic measures for thyroid hormone related diseases [13]. Most of the relevant components involved in TH biosynthesis, storage, secretion, systemic distribution, cellular uptake, metabolism and action have been identified by various “-omics” approaches.

### 1.2. Trace Element Uptake by Thyrocytes

The thyroid accumulates the essential trace element iodine in its inorganic anion form, iodide, but not other iodinated molecules or molecular iodine (I_2_). I_2_ easily sublimates, is volatile and strongly oxidizing. I_2_-containing (alcoholic) solutions may be used for drinking water disinfection or as skin and mucosal antiseptics and will oxidize unsaturated lipids under the release of iodide. The cloning, functional characterization and recent identification of the structure of the sodium-iodide symporter (NIS) provides detailed mechanistic insight into the efficient process of iodide accumulation across the basolateral membrane of thyrocytes against an electrochemical gradient [14,15]. Initially, an energy dependent ouabain-sensitive ‘iodide pump’ was proposed to mediate iodide uptake. However, the driving force for the electrogenic NIS symporter, which cotransports one iodide anion with two sodium cations, is provided by the ATP-dependent sodium–potassium ATPase of the basolateral membrane. NIS preferentially transports voluminous anions such as iodide, but not the other halide anions, as well as large oxyanions such as pertechnate, which is used for diagnostic purposes, and perchlorate, a clinically relevant competitor of NIS function. Iodide accumulation by the thyroid gland requires an efficient function of the iodide oxidation and TG organification and storage processes in the follicular lumen, as indicated by the absence of iodide accumulation in other cells and tissues, which also express NIS (albeit at a much lower level than thyrocytes) but lack the ‘thyroxisome’ enzymes and structure. Currently, no details are known about the uptake mechanisms involved in Se accumulation by thyroid follicles. Inorganic selenite or selenate are transported across membranes by sulfate and sulfite anion carriers in other cells [16,17] but no Se-specific transporters are known. Whether and to what extent inorganic selenite, SeMet or other small molecular Se compounds [18] reach thyrocytes remains to be studied. The high Se content of the thyroid is probably due to the expression of most selenoproteins in this “thyroxisomal TG factory” (see below). Iron uptake and its possible storage by thyroid follicles have not been studied apart from observations on the consequences of iron deficiency which impairs the function of the hemoprotein TPO and TH biosynthesis (see below, [12]).

### 1.3. Gene Variants Affecting the Thyroid Hormone System

Disorders of handling iodide along its path to TH, which impair the functions of TSHR, NIS, TG, TPO, DUOX/DUOXA2 and IYD, are known [19]. Similarly, (inherited) genetic disorders and variants of the selenoproteins required for TH biosynthesis and metabolism as well as the protection of the H_2_O_2_-exposed thyroid follicular structure have been identified [20]. Such alterations modify the sensitivity of the hypothalamus–pituitary–thyroid–periphery (HPTP) axis to its negative feedback regulation by TH and its adaptive response to nutritional, environmental, pharmacological and disease-related challenges. Genetic disorders affecting iron metabolism, transport, storage and cellular handling (e.g., β-Thalassemia) also disturb TH status, and diagnostic procedures for TH determination [21] and the adverse impact of hemochromatosis on thyroid function has been known since 1936 [22].

Most of these observations made in clinical studies and patient care have been substantiated by preclinical studies using dedicated animal models as well as cellular and molecular studies which enable the drawing of solid, intervention-based mechanistic conclusions and the establishment of clearcut cause–effect relationships with high clinical relevance, considering that the whole THS, its regulation and its individual components have been highly conserved along human ontogeny and phylogeny [23]. Such crucial information now receives further support from carefully planned and implemented epidemiological as well as prospective studies of sufficient power, linking clinical observations with nutritional and environmental exposure data. This approach is especially important when multifactorial changes in the development and progress of TH-related diseases tarnish the picture, and the effect sizes of the perturbations are small as, e.g., observed for the endocrine-disrupting chemicals affecting the thyroid gland and the whole THS in humans.

## 2. Thyroid Function and Iodine Status

The element iodine was more or less simultaneously discovered around 1811 by Bernard Courtois, Louis-Joseph Gay-Lussac and Sir Humphry Davy, and an adult healthy thyroid gland contains around 12–16 mg (0.5–1 mg iodine/g thyroid) [24]. Most foods and beverages have a low iodine content in their native state, while seafood, certain brown algae (‘kelp’), sea fish and seaweed as well as eggs, milk products, bread and food prepared with iodinated salt are the major sources of nutritional intake.

### Iodine Deficiency

Relationships between iodine supply and disorders of thyroid function, such as the evolution of endemic goiters and the occurrence of congenital hypothyroidism (formerly denominated under various categories of ‘cretinism’) at inadequate iodine intake, were observed especially in mountainous regions where the soil content of trace elements is low due to washouts during glacial periods. Nevertheless, these ideas also met strong opposition from highly influential scientists such as Virchow, who favored the then popular ‘miasm’ hypothesis to explain goitrogenesis and the development of ‘cretinism’. In his view, not iodine deficiency, as proposed in Switzerland and France, but ‘bad odors’, poor nutrition, housing, social status and related adverse environmental factors caused ‘cretinism’ [25]. Obviously, these factors will contribute to this multifactorial process, but will have a marginal role provided that iodine supply is adequate. The additional consumption of goitrogens contained in some varieties of plant-derived (vegetarian) food might exacerbate the adverse condition.

Recent EU-funded surveys revealed a sobering impression of the European iodine status, indicating that there is still a very long way to go to reach the goal of “Euthyroid: making Europe smarter with iodine” [26] (https://www.ign.org/newsletter/idd_feb16_euthyroid.pdf, accessed on 6 February 2023). While school children still obtain an adequate iodine supply, the situation in the adult population is still inadequate and deficient for pregnant women in several EU countries [27]. Obviously, the voluntary use of iodinated table salt in many countries, decreasing use of iodinated salt in (semi-)commercial food production, decreasing consumption of table salt for other medical reasons, and too-low iodine content in the form of KIO_3_ in table salt has contributed to this situation. According to WHO data, 60% of the European population do not reach the lower limit of urinary iodine excretion (100 μg/L), which corresponds 40 Mio children and more than 430 Mio adults [4,28] (http://whqlibdoc.who.int/publications/2007/9789241595827_eng.pdf; https://data.unicef.org/topic/nutrition/iodine/, accessed on 6 February 2023). In European countries with the voluntary use of iodinated salt (e.g., Germany), only in two of the nine countries do pregnant women reach a urinary iodine excretion higher than 100 (μg/L), which indicates a clear risk of impaired brain development and intelligence quotient (IQ) losses of up to 15 points [29]. While universal salt iodination programs during the last few decades have been very successful in decreasing goiter rate and the number of newborns affected by iodine deficiency disorders (IDD) in many regions of the world [28], around 5 Mio newborns will still be affected by IDD, impaired cognitive development, equal opportunities and a life-long loss of productivity [30]. Whether additional complications will arise related to concurrent disbalances of selenium and iron intake needs to be studied.

## 3. Thyroid Function and Selenium Status

The second essential trace element for the TH system, selenium, was discovered by the Swedish chemist J.J. Berzelius in 1817 [31]. The human body contains around 14 mg of Se. The recommended intake is 150–200 µg/d and the no adverse effect level (NOAEL) for human uptake is 800 µg/d. 

### 3.1. Selenium Deficiency

Se entered the thyroid stage 35 years ago when the first interactions between nutritional I and Se supply were observed in regions with endemic goiter and congenital hypothyroidism in Central Africa and the Himalayas [32]. Remarkably, two different forms of congenital hypothyroidism (myxedematous vs. neurological ‘cretinism’) were characterized and associated with the time-points of maternal and fetal iodine and (combined) Se deficiencies. As long as the maternal T4 supply for the developing fetus is adequate during pregnancy, the myxedematous form of congenital hypothyroidism may however develop in the case of deficient iodine supply during late pregnancy and the neonatal period. Concomitant Se deficiency may exaggerate the emergence of the myxedematous form of congenital hypothyroidism. Neurological ‘cretinism’ may develop with maternal iodine deficiency and subsequent hypothyroidism during the first trimester of pregnancy and later on [33]. The major mental and physically impairments of both forms of congenital hypothyroidism are irreversible.

Selenoproteins scavenge the excess H_2_O_2_ produced by the angio-follicular units of the thyroid, thus avoiding the local production of and cellular damage by reactive oxygen (ROS) and nitrogen (NOS) species [34]. Such damage might also be caused by lymphocytic infiltration of the thyroid gland, a cytological marker of Hashimoto’s thyroiditis, which affects up to one third of women in their reproductive and postmenopausal age and leads to the irreversible long-term destruction of functional thyroid tissue and fibrosis with the consequence of hypothyroidism [35]. This is the main reason why L-Thyroxine ranks among the top ten medically prescribed drugs worldwide.

Associations between nutritional Se intake, serum Se status and the prevalence of thyroid-hormone-related diseases had been reported during the last three decades, mainly in small observational or interventional studies in various regions, subpopulations and study groups with different basal Se intakes [11,36]. Only a few large epidemiological, observational or prospective studies are available [13], but these support the close link between inadequate Se status and the manifestation of TH-system-related disease, which is also strongly substantiated by animal experimental and in vitro studies providing clearcut cause–effect relationships.

### 3.2. Se Status and Thyroid Hormone Concentrations

Remarkable associations between dietary Se intake, based on two 24 h dietary recall interviews, and serum thyroid hormone concentrations have recently been reported by a NHANES study with more than 5500 US adult participants. Increased Se intake was negatively correlated with total T4 and the total T4/total T3 ratio, especially in males with adequate iodine intake. Authors discussed that in females, the stronger effect of sex steroid status might obliterate such relationships and that adequate Se intake might stimulate T3 formation by the deiodinase selenoproteins [37]. An inverse correlation between Se intake and subclinical hypothyroidismwas demonstrated in a large study cohort of 14,283 adult employees of both genders in Brazil [38]. In a prospective double-blind study of euthyroid subjects (60–74 years old) receiving a selenium–yeast-based supplementation (100, 200, or 300 µg Se/day for 5 years), decreases in serum TSH and fT4 concentrations but no changes in fT3 or the FT3/fT4 ratio were observed, indicating a dose-dependent effect on thyroid function even in healthy individuals [39]. A recently published prospective observational, but not interventional, cohort study observed an increased incidence of Hashimoto’s thyroiditis after 6 years of follow-up in those 1254 individuals with low Se status as documented by informative biomarkers [40]. This study was performed in the same area where a previous analysis revealed a higher prevalence of clinically relevant thyroid disorders in a region with low-selenium intake compared to a population residing in a near neighboring county with the same genetic and cultural background and similar nutritional, living and working conditions and comparable dietary patterns but adequate-selenium status [41]. A recent evaluation of NHANES data [42] of dietary Se intake in almost 9000 US adults during 2007–2012 revealed an inverse relationship between Hashimoto’s thyroiditis and serum TPO antibodies. This affirms the link observed in various small studies and lending epidemiological support for a strategy preventing the manifestation of autoimmune thyroiditis by securing an adequate nutritional Se intake, especially in adult females. Nevertheless, prospective, adequately powered clinical trials for patients with thyroid diseases receiving Se supplementation are urgently needed. 

### 3.3. Selenium Deficiency and Congenital Hypothyroidism

Under conditions of inadequate iodine intake, Se supplementation might be counterproductive and iodine deficiency needs to be remediated to an adequate intake before Se compounds are administered. This has been documented by an inadvertent observation during the initial attempts to restore essential trace element status in a population with too-low nutritional iodine and Se intake in Central Africa [43]. Among the treated individuals, a patient with the myxedematous form of ‘cretinism’ also received Se supplementation. In this case, Se supplementation for six months resulted in severe hypothyroidism with very low total T4 and highly elevated TSH. Probably, this small fibrous thyroid gland with a low iodine intake and accelerated iodine metabolism might rapidly lose its limited iodine stores. Extrathyroidal deiodinases, previously lacking selenium supply, will be synthetized and become activated by Se supplementation to deiodinate circulating TH, presumably leading to renal and fecal iodine losses under this peculiar condition and exaggerating the thyroidal iodine deficiency. Thus, before Se supplementation, an adequate iodine status must be secured. Support for this reasoning has been documented by experimental studies in rats, which revealed that high iodine supplementation in Se deficient animals induced severe TGF-β-mediated fibrosis of their thyroid gland, which can be prevented by the earlier restoration of an adequate Se supply [44]. A sufficient Se supply can prevent the adverse thyroidal effects of excessive iodine intake, probably because thyroidal selenoproteins adequately translated under conditions of sufficient Se intake can protect the gland from the ROS and NOS generated by excessive iodine intake [45]. Before any preventive and interventive Se supplementation will be initiated, an adequate iodine status should be secured in order to avoid any undesirable loss of iodinated TH from the system. 

A high Se content in mouse thyroids was even found under conditions of very low Se intake (0.2 ppm) [46]. While other tissues are already depleted as, e.g., indicated by their very low expression of selenoproteins, Se content in the thyroid still remains in the regular range.

## 4. Thyroid Function and Iron Status

The third essential trace element required for regular TH biosynthesis and function is iron. The body of an adult human contains about 4 g (0.005% body weight) of iron, mostly in hemoglobin and myoglobin, but also in a variety of cytochromes and other hemoproteins containing Fe central atom of the prosthetic groups in their active site, such as thyroperoxidase (TPO, EC 1.11.1.1-14). TPO, solely expressed by thyrocytes and related to myeloperoxidase and lactoperoxidase, is the multifunctional key enzyme of TH biosynthesis [47]. It catalyzes the sequence of (i) H_2_O_2_-dependent oxidative reactions of iodide oxidation to an iodonium intermediate, followed by (ii) the iodination of selected tyrosyl-residues of TG, the protein enabling synthesis, storage and secretion of TH, and finally, the coupling of selected diiodo-tyrosine (DIT) residues of TG to T4, or of one DIT and one monoiodo-tyrosine (MIT) to T3. The heterodimeric complex of dual oxidase (DUOX, EC:1.6.3.1) and its maturation factor, DUOXA2, generates the required H_2_O_2_ in a NADPH-dependent reaction maintained by the pentose phosphate cycle [48]. These proteins are organized as ‘thyroxisome’ [49] at the apical surface of thyrocytes facing the colloidal lumen, into which dimeric TG is secreted, and stored after its iodination. All of these steps of iodide uptake and utilization for TH biosynthesis, storage and secretion are stimulated by the pituitary hormone activating its TSH receptor (TSHR), a G-protein-coupled receptor in the basolateral membrane of thyrocytes [50]. The details of the TPO-catalyzed reactions are not fully understood.

### Iron Deficiency and Excess

Iron deficiency and anemia are associated with hypothyroidism probably due to the impaired biosynthesis of the hemoprotein TPO, but the central feedback regulation of the HPTP axis is also disturbed [51]. On the other hand, patients suffering from thalassemia major, one of the most frequent hereditary diseases affecting erythropoiesis and hemoglobin synthesis, require multiple blood transfusions. These increase iron content in the blood, which requires the subsequent chelation of liberated Fe. Many of these patients develop (subclinical) hypothyroidism [21]. The restoration of adequate serum ferritin concentrations, a biomarker for the body’s iron stores, supports the recovery of the regular function of the TH system and its serum markers TSH and fT4 [52]. This is of high relevance in iron-deficient pregnant women [53]. However, the interrelationship between Fe and TH is bidirectional as TH directly stimulate erythyropoiesis, predominantly via the TR-alpha receptor [54,55]. Fe supplements and improvement in iron status in areas with endemic deficiency facilitate iodide utilization for TH biosynthesis, as demonstrated in children [12] as well as for anemic (mainly female) patients [56]. The development of autoimmune thyroiditis, mostly affecting women and characterized by autoantibodies against TPO circulating in the blood, aggravates this condition. Combined treatment with T4 and sufficiently bioavailable iron supplements might be needed and clinical studies demonstrated better effects than those accomplished by each of the factors alone [57,58].

## 5. Selenoproteins

**Figure 2:**
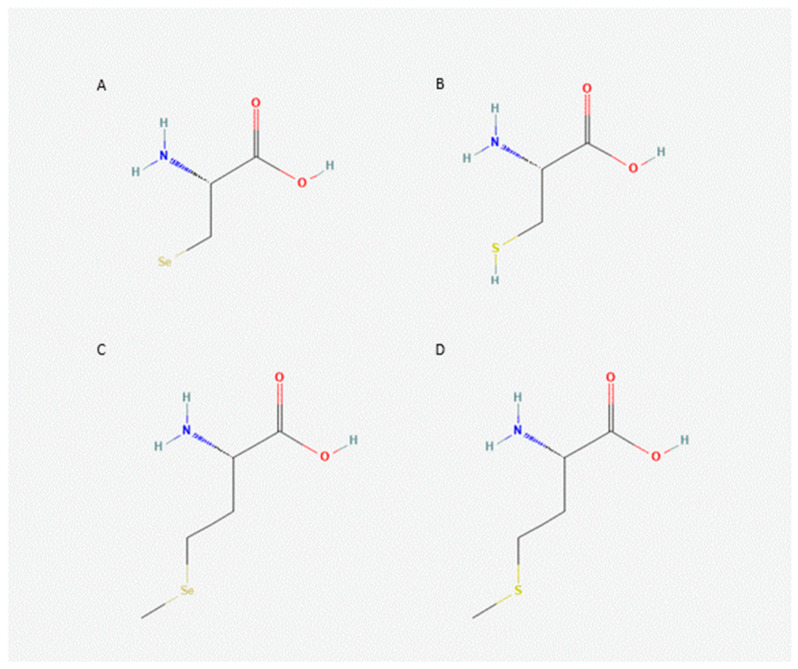
Structures of natural selenoamino acids, selenocysteine ((**A**), SeCys; CID 6326983) and selenomethionine ((**C**), SeMet; CID 105024), and their sulfur homologues, cysteine ((**B**), Cys; CID 5862) and methionine ((**D**), Met; CID 6137). Structures obtained from Pubchem National Center for Biotechnology Information (2023), https://pubchem.ncbi.nlm.nih.gov, accessed on 7 February 2023.

**Figure 3:**
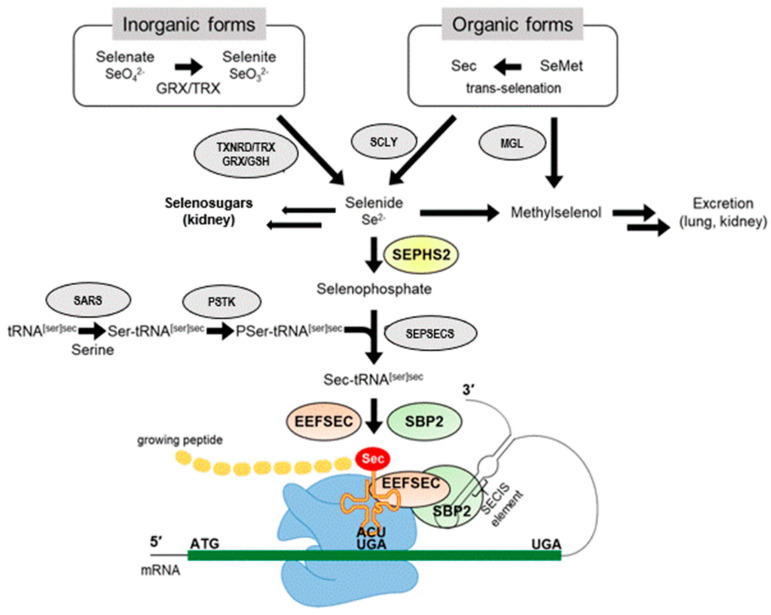
Nutritional sources of selenium and their utilization for biosynthesis of selenocysteine-containing proteins. Abbreviations: SeMet, selenomethionine; Sec, selenocysteine; GRX, glutathione reductase; TRX, thioredoxin; TXNRD, thioredoxin reductase; GSH, glutathione; MGL, methionine gamma-lyase; SCLY, selenocysteine lyase; SEPHS2, selenophosphate synthetase 2; SARS, seryl-tRNA synthetase; PSTK, phosphoseryl(Sep)-tRNA kinase; SEPSECS, Sep-tRNA:Sec-tRNA synthase; EEFSEC, Sec-specific eukaryotic elongation factor; SBP2, SECIS binding protein 2. Adapted from [69], Kang D, et al. (2020).

The essential trace element Se is incorporated into proteins either as selenomethionine (SeMet) or as selenoysteine (SeCys, Sec), the 21st proteinogenic amino acid [59] (Figure 2). The latter pathway requires a three-stage process, catalyzed by selenophosphate synthase organifying Se (probably in the form of Se^−2^ or H_2_Se) in an ATP-dependent process to activated selenophosphate, which phosphorylates seryl-tRNA synthetase (Figure 3). The enzyme Sep (O-Phosphoserine) tRNA:Sec (Selenocysteine) tRNA Synthase, encoded by the *SEPSECS* gene, converts this phosphorylated tRNA to selenocysteinyl-tRNA(Ser)Sec using the activated selenophosphate. This Se-containing aminoacyl-tRNA can then occupies a ribosomal free A-site for the cotranslational incorporation of Sec into the nascent polypeptide chain encoded by a selenoprotein mRNA. UGA triplets in these transcripts encode the cotranslational incorporation of Sec if a stable hairpin loop structure, the selenocysteine insertion sequence (SECIS-element), is located downstream of the UGA in the 3′-noncoding region of a selenoprotein mRNA. The SECIS element is recognized by SECIS-binding protein 2, which then recruits elongation factor 2 to steer the incorporation of Sec at the UGA codon position. In the absence of Se or any of these factors, a UGA codon will lead to ribosome pausing and the premature termination of the protein chain [60,61]. In contrast to the complex mechanism of Sec biosynthesis and its incorporation into selenoproteins, which requires several cis- and trans-acting elements of the translation machinery, the second selenium-containing amino acid SeMet originates from the stochastic incorporation of Se, instead of regular sulfur, during the biosynthesis of methionine. SeMet is randomly incorporated into proteins instead of S-containing methionine according to its abundance in the cells [62]. While SeMet can be recycled for de novo incorporation as SeMet into proteins, Sec is released by proteolysis from selenoproteins and then degraded by Sec β-lyase which releases selenide that can be metabolized for its excretion or again undergo the complex tightly regulated biosynthesis pathway required for Sec-containing proteins [63]. The term selenoproteins therefore is only used for the latter group, which exert specific (enzymatic or structural) functions for cellular metabolism and homeostasis depending on the highly reactive redox-active properties of Sec in their active sites and not executed by the regular S-containing cysteine residues in proteins. In contrast, no specific functions are known for SeMet in proteins which would not also be exerted by regular S-methionine. However, SeMet residues in proteins can be much more easier oxidized than S-methionine and this may lead to a loss of protein function or altered antigenicity [64,65]. Methionine is one of the most easiest oxidized amino acids and two mammalian enzymes (methionine sulfoxide reductases, Msr) are known to reduce methionine sulfoxide and hereby restore protein function [66]. One of these two enzymes (MsrB, EC:1.8.4.12) is also one of the selenoproteins encoded by the 25 selenoprotein genes in humans. A high nutritional Se intake, especially in the form of SeMet, selenized yeast (mainly containing SeMet), and various inorganic or organic low-molecular-weight Se-containing compounds can be readily incorporated into SeMet and corresponding proteins without any limit and thus accumulate in a protein-bound Se-reservoir (devoid of a specific biological function) of the organism [67]. In contrast, the cotranslational incorporation mechanism of Sec into specific proteins is restricted by their transcriptional and translational control, limiting any Se-accumulation in the body. Only selenite, after its reduction to Se^2^- or Se^2−^ released from other completely reduced Se-compounds, is available for the regulated biosynthesis of Sec-containing selenoproteins. Altered Se status and the disturbed function of selenoproteins will have a major impact on the homeostasis of various metabolic, structural and regulatory aspects of a living individual, eventually jeopardizing its health and survival [68].

### 5.1. Selenoproteins of the Thyroid

**Figure 4:**
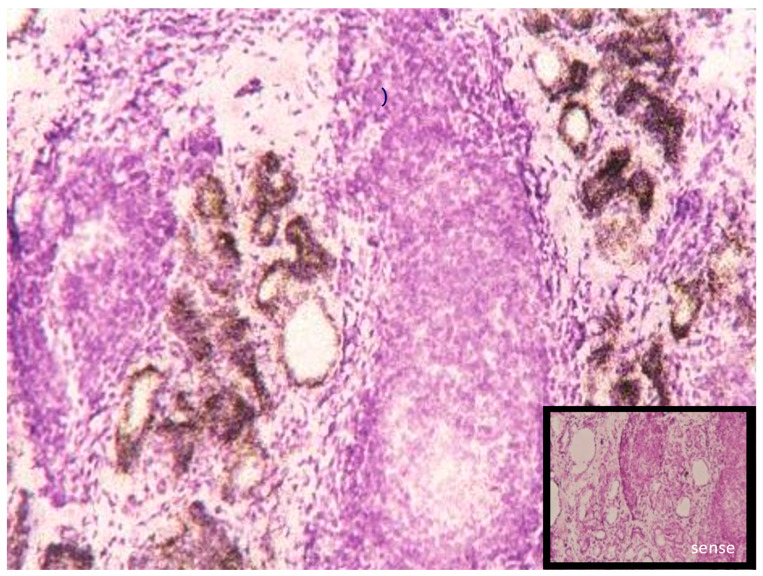
Expression of pGPx (Gpx3) mRNA detected in in situ hybridization (12 µm cryosection) of a human thyroid gland specimen, thyroidectomized for severe Hashimoto’s thyroiditis. Residual angiofollicular units stained for pGPx (brown); H&E stain of infiltrating lymphocytes; insert: negative control staining with an antisense pGPx probe. (Reproduced with permission from Figure 1C [6], Schmutzler C. et al. *Biol. Chem.* (2007), *388*, 1053–1059; DOI:10.1515/BC.2007.122).

The human thyroid gland, similar to other endocrine tissue, is characterized by one of the highest Se contents per gram of tissue in a healthy individual consuming a balanced nutrition [70]. This Se content is bioavailable, while some tissues (kidney, pituitary, etc.) assumed to be rich in selenide deposits of heavy metal selenides (HgSe, CdSe, PbSe) have been described which cannot be mobilized anymore [71,72]. The mechanisms of Se uptake into thyroid follicular cells are unknown. Most of the selenoproteins known are found to be expressed in the thyroid at the transcriptional and protein level [6,34]. Members of the GPX and TXNRD families probably cope with the challenge of the life-long redox control and antioxidative defense against H_2_O_2_-. DIO1 and DIO2 are highly expressed in a species-dependent pattern, as well as stimulated by TSH- or Graves’-IgG-dependent cAMP-mediated signaling [73,74]. In addition, hyperthyroidism enhances the expression of DIO1 and local T3 production resulting in a higher T3/T4 ratio secreted by the gland, similar to that under the conditions of iodine deficiency where there is already relatively more T3 contained in TG compared to a regular iodine status [75]. TG is the main protein produced by follicles, and due to its enormous homodimer size (2 × 330,000 kDa), complex glycoprotein structure, secretion, deposition, polymerization and mobilization, processes in the follicular colloid require a battery of quality control mechanisms, including those exerted by members of the endoplasmic-reticulum-resident selenoproteins [76] typically involved in ribosomal biosynthesis and the posttranslational modification of proteins. Currently, it is not yet clear which of the selenoproteins are crucial for proper TG biosynthesis and whether their inadequate expression or function in the angio-follicular units of the thyroid gland may contribute to the development or perpetuation of autoimmune thyroid diseases or thyroid cancer [77]. In sections of ‘normal’ human thyroid tissue, adjacent to surgically resected tumor tissue GPx1, SelM, SelS are highly expressed at the protein level; pGPx (GPX3) and SELENOP were even found in the colloid lumen, while DIO2 signals were mainly located in the microvascular system and no DIO3 expression has been reported in thyroid glands. During the in situ hybridization of tissue from a gland affected by autoimmune thyroiditis (M. Hashimoto), the very strong expression of GPx3 was seen in residual follicular structures whose infiltrating lymphocytes and fibrous structure were devoid of any GPx3 signal (Figure 4).

### 5.2. Genetic Models Manipulating Cellular and Systemic Selenium Availability via Selenoprotein Biosynthesis

Significant insight into the complex network of incorporation, retention and release of Se by specific selenoproteins has been obtained from the systematic manipulation of the expression of selenoproteins using genetic models in mice where specific selenoprotein genes have been inactivated, mutated or transgenically expressed either globally or conditionally in a cell-/tissue-specific manner [78,79]. The recent applications of CRISPR-Cas9 methodology enormously accelerated the stem-cell-based cloning approaches and generation of cellular and in vivo mouse models. Two more broad approaches are (i) the inactivation of the *Trsp* gene [80], which completely prevents the biosynthesis of all selenoprotein, (ii) while *Scly* inactivation [81] blocks the cellular recycling of Se/selenide contained in the Sec of selenoproteins. The conditional inactivation of *Sbp2* in mice results in the markedly decreased expression and/or activity of several selenoproteins in a tissue-specific manner and a thyroid hormone pattern in serum (high T4, rT3 and TSH, regular T3) mimicking the phenotype of such mutations in humans [82]. Some of the genetic manipulations replaced the UGA codon with those of the functionally related cysteine or the sterically similar serine in order to avoid major structural changes in the polypeptide chain. These studies significantly contributed to the revealing of the impact of disturbances of UGA decoding and Sec insertion machinery on several rare diseases and to the delineation of the molecular and mechanistic details of the biochemistry of selenoproteins [83]. Nevertheless, several open questions remain as exemplified by the approach to better understand the role of Se and selenoproteins for the THS.

A complete inactivation of selenoprotein synthesis in mouse thyroid follicular epithelial cells, intended to test their presumed antioxidative function during the life-long production of H_2_O_2_, did not result in the expected destructive process of the follicular organization and function of the thyroid [84]. Even challenges to the gland produced by severe iodine deficiency, after feeding these mutant mice a low iodine–perchlorate diet, resulting in decreased T4, elevated TSH and regular T3 (the latter was expected), did not destroy their thyroid glands. While increased lipid peroxidation and nitrosative stress was visible from immunohistochemistry, the morphology of the gland was unchanged and neither signs of fibrosis nor lymphocytic infiltration, typical for autoimmune thyroiditis in patients, were found. The activities of selenoproteins Dio1 and Gpx were strongly reduced. Thyroidal selenoprotein expression is obviously not essential for maintaining follicular integrity but may be of advantage for the prevention of major oxidative damage. Several aspects still need to be clarified: (i) Is this mouse model reliably recapitulating the human scenario of development of autoimmune thyroid diseases, known to have a strong component of genetic predisposition and gender dependence? [85,86]; (ii) Does the development of autoimmune thyroid diseases (AITD) require a long term disbalance of thyroid function and its interaction with an activated immune system? (iii) Might relatively short-lived mice not adequately mimic such a disease history, although various mouse models have been developed which replicate many but not all aspects of human AITD? (iv) Is the protective function of adequate Se status and selenoproteins located in the endothelial or stromal cells but not in the epithelial thyrocytes? (v) Do other non-selenoprotein antioxidative enzymes inactivating H_2_O_2_ (catalase) and ROS derived therefrom compensate (superoxide dismutase, SOD) for absent selenoproteins?

Currently, it is not clear how and to what extent Se is transported to the thyroid and which factors contribute to its accumulation, which might solely be caused by the high expression levels of various selenoproteins [6]. The global genetic inactivation of Selenop expression in mice neither affected thyroid morphology, nor the function and expression of those selenoproteins analyzed in this tissue, although serum Se status was markedly decreased [87]. This observation supported previous long-term nutritional Se withdrawal experiments in rodents, which left the thyroid, several other endocrine glands and testes unaffected [88,89].

### 5.3. Selenoproteins Relevant to the THS

Se Status and selenoprotein functions are not only relevant to TH biosynthesis but also essential for TH activation and inactivation as well as for iodine homeostasis and excretion. No information is yet available whether TH distribution in the blood [90] or TH transport across cellular membranes is affected by Se status. The multifunctional TH distributor protein transthyretin, originally evolved from the family of 5-hydroxyisourate hydrolases (HIUHase), contains zinc which is essential for T3 binding in fish but not for T4 and T3 binding in humans [91]. No transthyretin interactions with Se or I status have been reported. 

The identification of DIO1 as a selenoprotein, the subsequent cloning of the DIO1 gene, quickly followed by those of DIO2 and DIO3, opened a new era for the better understanding of TH homeostasis and the nutritional impact of trace elements on the TH system. Subsequent to the GPX family, the three DIOs were the second enzyme group whose function and reaction mechanisms critically depend on Sec in their active sites [7]. The TXNRD gene family, also coding for selenoproteins, was the third group of Sec-containing enzymes catalyzing the essential redox reactions involved in the regulation of cell differentiation, proliferation and antioxidative redox control, also in the context of the adequate function of the THS [34,92]. Cellular and animal model studies creating experimental conditions of severe Se deficiency, restoration and excess, established the clear dependence of DIO isozyme translation and function on cellular Se availability and nutritional Se status. The observed responses were cell-/tissue specific and supported the hypothesis of a tissue-specific hierarchy of Se incorporation into various selenoproteins [93]. Similarly, a hierarchy is also found between selenoproteins with, e.g., Gpx4, Dios and some of the selenoproteins assisting with protein folding. Intracellular DIOs are crucial for the pre-receptor control of local T3-ligand availability to the T3 receptors, which act as T3-ligand-dependent transcription factors modulating the expression of TH-responsive genes in target cells of TH action [10]. 

An inadequate Se status impairs DIO function, enhances the formation of TH conjugates (especially sulfates) [94,95], prevents their excretion, altogether favoring enterohepatic recycling and thereby decreasing iodide loss in feces and urine. TH conjugates, inactive as ligands at the T3-receptors, participate in the enterohepatic recycling of TH and may represent a reservoir for TH mobilization after the enzymatic hydrolysis of their sulfates or glucuronides. Whether the (transient) storage of TH conjugates such as T4-glucuronide in tissue reservoirs such as the kidneys is functionally relevant and regulated, needs to be studied in more detail [96].

The T3-receptor protein family, encoded by two different genes (*THRA, THRB*) and occurring in various protein isoforms and isotypes depending on the tissue-specific splicing of their transcripts, is sensitive to a redox control similar to the other 49 members of the c-erbA family of ligand-modulated (nuclear) receptors [97]. No specific alterations in the expression and function of TRs have been reported with respect to either the Se status and availability or the dependence on the iodine or iron status under (patho-) physiologically relevant in vivo conditions. 

Obviously, drastic manipulations of in vitro conditions, such as applying high or pharmacological concentrations of Se-, I- or Fe-compounds which may disrupt TR function in cell models or in vitro paradigms, are too frequently advertised and used in toxicology-oriented studies claiming in vivo relevance on a “new approach methodology” (NAM) in vitro basis [98]. Even if Se and selenoproteins play a major role in TH synthesis, metabolism and action, Se deficiency and excess will not exert immediate adverse effects and disturbances of the THS, which is highly essential for development (especially of the brain), cell differentiation and growth as well as the homeostasis of energy metabolism, anabolic and catabolic processes, because synthesis, metabolism and action of TH occur in a much lower nano- to femtomolar concentration range compared to, e.g., the reactions of intermediary metabolism. Se utilized and stored in the major selenoprotein families involved in such high-turnover reactions can easily be mobilized to supply the very small Se demands by all DIOs expressed in the whole intact organism. Alone, the mobilization of Se from hepatic GPX1 turnover under conditions of nutritional Se shortage or deficiency, 1000 times more abundant compared to hepatic DIO1, would probably provide sufficient Se for the de novo synthesis of DIOs and other crucial selenoenzymes (e.g., GPX4, TXNRD; etc.), ranking similarly high in the UGA-decoding and Sec-translation hierarchy of selenoproteins [99,100].

While cellular and animal experimental data might indicate the major adverse effects of severe Se deficiency and excess on the THS, conditions occurring in human healthy life and diseases might be less extreme and also prevented by multiple compensatory mechanisms. Exceptions obviously will be seen in case of monogenetic diseases affecting components of the Se system [101], or clinical conditions such as long-term parenteral nutrition devoid of adequate Se supplementation or intoxications by excessive doses of Se compounds when the essential trace element Se is mistaken for a mass mineral element such as Na or Ca or for compounds such as glucose or fatty acids. Nevertheless, many clinically relevant conditions of inadequate nutritional Se intake have been identified, which result in the limited performance of functional selenoproteins and enzymes, and thus should be avoided by preventive nutritive measures or treated with adequate supplemental or pharmaceutical Se preparations, when deficiencies are ascertained by proper methods, parameters or biomarkers, and not only on the presumed suspicion of Se imbalance [102]. 

## 6. Bio-Availability and Bioactivity of Se and Se-Containing Compounds

While natural nutritional sources of Se intake are mainly based on Sec as a constituent of selenoproteins and SeMet-containing proteins, commercially available Se supplements come in various inorganic and organic forms. This variability is one reason for the divergence in clinical (pilot) studies which mainly are underpowered, short and lack clear information on basal Se status and the detailed speciation of the Se content of the preparations used [18,103]. Recently, novel forms of Se supplements entered the OTC, and the online and internet market of Se compounds and were appraised as next-generation Se supplements less toxic than previous traditional inorganic forms (selenite or selenate) because they apparently bypass classical cellular uptake mechanisms for inorganic anions or the amino acids Sec and SeMet [104,105,106,107,108]. These cellular ‘mini-taxis’, assumed to deliver the essential trace element bound to various bioactive carrier and coating molecules, are rapidly absorbed by cell membranes and may prevent interference with the cellular redox control system rapidly metabolizing selenite and selenate. Se nanoparticles mostly contain elemental Se^0^ bound to various polymeric carriers composed of inorganic or organic (charged and/or coated) building blocks. Whether any elemental Se^0^, an inert metal, will be bioavailable at all and subsequently incorporated into selenoproteins, which exert most of the biological (not pharmacological or toxic) effects of the essential trace element, seems to be unclear. If Se^0^ will not be reduced to Se Se^−2^, no UGA-codon-dependent incorporation into selenoproteins may occur, and if not oxidized to selenite Se^+4^ or selenate Se^+6^, no stochastic incorporation into SeMet and SeMet-containing proteins will occur, resulting in a higher retention of Se in the protein pool of the organism. Some limited information has been reported on microbial reduction of elemental Se^0^. Whether and to what extent this may be accomplished by the gut microbiome needs to be studied before such (adjuvant) Se supplements can be declared as safe and relevant.

A similar Se speciation issue is encountered when Se tissue contents were analyzed under regular nutritional intake and after the consumption of Se supplements or Se-containing drugs. Se^2-^ formed in vivo from such precursors or released by Scly, catalyzing Sec metabolism, rapidly interacted with bivalent heavy metal cations such as Hg, Cd, Pb, etc., forming virtually insoluble selenides deposited at their site of formation, mainly in the kidney, endocrine tissues rich in selenoproteins, and in the brain. The solubility products of these heavy metal selenides is below 10^−55^ mol^2^·L^−2^ and they will probably “never” be redissolved again in a living organism. These also contribute to the high heavy metal content of seafood and sea fish [109,110], leading to the probably inappropriate recommendation for pregnant and lactating women to avoid these nutrients rich in unsaturated fatty acids, due to the fear of heavy metal (esp. Hg, Cd, Pb) intoxication of the mothers and their developing children! However, an adequate Se intake, closely associated with iodine intake in the form of sea fish and seafood, will efficiently protect mothers, fetuses, newborns, and their brains and thyroids from serious heavy metal damage.

This simple inorganic chemistry is mostly neglected if fancy high-tech labs with expensive machines and methods perform and communicate their (alarming) analytics and avoid specifying exactly which chemical species (e.g., methylmercury, Hg^2+^, soluble ion or “innocent” HgSe or HgS) is contained in a sample!

## 7. Conclusions and Outlook

Although the crucial role of the adequate supply of the essential trace element iodine for regular thyroid function and TH biosynthesis has been known for more than a century, no successful worldwide measures have yet been achieved which have resulted in a globally sufficient nutritional iodine intake or the cheap prevention of the simply avoidable fatal medical and societal impact of iodine deficiency and its multiple sequelae [28]. Most of the genetic, molecular, biochemical and medically relevant features of the individual components of iodide uptake, utilization, and function of TH have been deciphered during the last few decades and enabled medical approaches in diagnosis, treatment and the monitoring of (causal) therapeutic procedures [111,112] (http://www.thyroid.org/thyroid-information/, accessed on 7 February 2023). However, adequate iodine availability and thyroidal processing to TH is not the only bottleneck ensuring a regular thyroid gland performance during fetal development and throughout life. Similarly limiting are the persistent nutritional availabilities of the two other essential trace elements, selenium and iron, both of which are required not only for biosynthesis of the iodine-containing TH by the gland but also for their systemic availability and action. Unlike iodine, which is ‘stockpiled’ for up to three months in the TG-containing colloid of the angiofollicular units of a healthy thyroid gland, both Se and Fe need to be constantly delivered via adequate balanced nutrition [13,56,113,114]. They are only retained in the human body in the form of Se- and Fe-containing proteins circulating in the blood or expressed as functional components, mainly as essential constituents of the cellular redox systems [115,116]. For both Se and Fe, systemic and cellular transport and storage proteins have evolved, but have a much lower reservoir function for these trace elements than thyroidal TG for iodine. 

Currently, our knowledge about thyroidal uptake, utilization and potential storage mechanisms for Se und Fe, and the interaction of such mechanisms with the thyroid utilization and homeostasis of the iodine system is lacking and thus purposeful basic and clinical research on these relevant interactions is urgently needed. Only limited experimental and clinical evidence for the need of a balanced supply of I and Fe on the one hand [12,52,113,114,117], and of I and Se on the other hand [43,113,118,119], has been reported. Thus, inadequate Fe status impairs the utilization of I, even if this element is sufficiently available. Whether excess Fe intake also leads to adverse effects on the thyroid gland and TH biosynthesis is unknown, and no information is available as to whether an inadequate I supply (excess or deficiency) adversely impacts on Fe homeostasis. 

More clear are the relationships between I and Se based on animal experimental data and some (epidemiological or interventional) clinical observations [44,120,121,122,123]. Consequences of mild I deficiency are less severe in the context of mild Se deficiency, probably due to lower TH metabolism and turnover via the deiodinase system. An increase in I supply under conditions of mild I deficiency unmasks an underlying Se deficiency. Thus, measures to improve nutritional I intake should be accompanied by adjusted increased nutritional Se (and Fe) supply in order to secure regular thyroid function and to avoid local adverse effects (such as oxidative damage, inflammation, fibrosis) of (too) high I availability for the angiofollicular units. Disbalances between trace element supply, local I utilization, TH synthesis and secretion have been observed under conditions of (genetically caused) congenital hypothyroidism, various autoimmune thyroid disease conditions and for thyroid cancer, both in humans but more so in appropriate animal experimental models mimicking such disease conditions [46,77,124]. The thoughtful translation of available knowledge gained from molecular, biochemical, in vitro and in vivo animal experimental studies to the human clinical practice still needs to be intensified.

## Data Availability

No new data were created or analyzed in this study. Data sharing is not applicable to this article.
